# Biostimulant Formulations and *Moringa oleifera* Extracts to Improve Yield, Quality, and Storability of Hydroponic Lettuce

**DOI:** 10.3390/molecules28010373

**Published:** 2023-01-02

**Authors:** Naouel Admane, Giuseppe Cavallo, Chahinez Hadjila, Maria Maddalena Cavalluzzi, Natalie Paola Rotondo, Antonio Salerno, Joseph Cannillo, Graziana Difonzo, Francesco Caponio, Antonio Ippolito, Giovanni Lentini, Simona Marianna Sanzani

**Affiliations:** 1 CIHEAM Bari, Via Ceglie 9, 70010 Valenzano, Italy; 2 Dipartimento di Farmacia—Scienze del Farmaco, Università degli Studi di Bari Aldo Moro, Via Orabona 4, 70125 Bari, Italy; 3 Forza Vitale, Via Castel del Monte, 194/C, 70033 Corato, Italy; 4 Dipartimento di Scienze del Suolo, della Pianta e degli Alimenti, Università degli Studi di Bari Aldo Moro, Via Amendola 165/A, 70126 Bari, Italy

**Keywords:** *Moringa oleifera*, protein hydrolysates, biostimulants, lettuce, hydroponic system

## Abstract

The urgent need to increase the sustainability of crop production has pushed the agricultural sector towards the use of biostimulants based on natural products. The current work aimed to determine whether the preharvest application of two commercial formulations, based on a Fabaceae enzymatic hydrolysate or a blend of nitrogen sources including fulvic acids, and two lab-made aqueous extracts from *Moringa oleifera* leaves (MLEs), could improve yield, quality, and storability of lettuce grown in a hydroponic system, as compared to an untreated control. Lettuce plants treated with the MLEs showed significantly improved quality parameters (leaf number, area, and color), total phenolic content and antioxidant activity, and resistance against the fungal pathogen *Botrytis cinerea*, comparable to that obtained with commercial formulates, particularly those based on the protein hydrolysate. A difference between the *M. oleifera* extracts was observed, probably due to the different compositions. Although further large-scale trials are needed, the tested MLEs seem a promising safe and effective preharvest means to improve lettuce agronomic and quality parameters and decrease susceptibility to rots.

## 1. Introduction

Ready-to-eat products have shown the most rapid expansion in production among vegetable products, even in developing countries [1]. Among the most widely cultivated vegetables are lettuce (*Lactuca sativa* L.) and chicory, accounting for over 27 million tons harvested worldwide in 2020 [2]. The reasons for this are the quick and year-round production, as well as the beneficial phytochemical and bioactive contents (vitamins, carotenoid, polyunsaturated fatty acids, antioxidants, etc.) [3]. Lettuce is one of the most cultivated leafy green vegetables in Italy, comprising, together with chicory, a production of 963.280 tons in 2021 [2]. Moreover, due to the conversion of many farms to the production and sale of ready-to-eat and fresh-cut salad mixes, lettuce cultivation has gained substantial economic importance in recent years owing to constant varietal and technical progress [4]. 

However, vegetable production is under pressure to raise yields due to the predicted increase in world population from 8 to 9.6 billion around 2050 [5], of which 70% will live in urban centers. Another major challenge posing a significant risk to the stability of production is climate change [6], which will stress the food supply chain by increasing chemical inputs [7]. Consequently, the demand for raw or minimally processed vegetables is rising, and food industries have been urged to utilize new cultivation systems and techniques to produce enough high-quality and safe food without detrimental effects to the environment or losses after processing or during cold storage. Food industries are also interested in increasing yield or growth rate to reduce costs and drive competitive pricing.

Nevertheless, the reliance on chemical inputs to increase production is also an issue due to the possible adverse effects on human and animal health and the environment due to inappropriate use, e.g., the nitrification of waterways [7]. In addition, the rising awareness of consumers on the importance of consuming sustainable, safe, and healthy foods [8] could be met by improving the quality of leafy vegetables with the use of natural biostimulants. According to the European Biostimulant Industry Council [9], plant biostimulants contain substance(s) and/or micro-organisms, which, in contact with the plants or the rhizosphere stimulate natural processes to enhance nutrient uptake, nutrient efficiency, tolerance to stresses, and crop quality. There are various sources of natural biostimulant products now available, including: (i) seaweed extracts, which boost plant germination, growth, and yield, while also enhancing biotic and abiotic stress tolerance [10]; (ii) microbial antagonists, which might consist of arbuscular mycorrhizal fungi and rhizobacteria [11]; (iii) protein hydrolysates (PHs), which are composed of peptides and amino acids derived from various animal or plant matrices [12]; and (iv) humic and fulvic acids [13]. Their action also includes an increase in carbon metabolism [14], and hormone activity [15], as well as physiological, biochemical, and anatomical changes such as the production of antioxidant enzymes, pigments, and secondary metabolites [16]. However, the beneficial plant effects may vary depending on plant species, cultivars, climatic circumstances, dosage, provenance, and timing of treatment [17]. In recent years, natural biostimulants have been increasingly integrated in a sustainable manner into high-value production systems (e.g., greenhouse production), improving productivity and quality, particularly under low input conditions [18]. Plant biostimulants provide a good fit with soilless systems since their purpose consists of improving nutrient use efficiency, tolerance to abiotic stress, quality traits and the availability of confined nutrients in soil or rhizosphere (EU Regulation 2019/1009). Indeed, soilless systems might respond to concerns about excessive land use, misuse of fertilizers and soil-borne diseases associated with intensive agriculture [19], as well as contributing to urban farming in an environmentally friendly and energy-efficient way [20]. 

Among natural biostimulants, moringa (*Moringa oleifera* Lam) leaf extracts (MLEs) attained massive attention due to their notable effect on plant productivity [21,22,23]. MLE applications proved to increase vegetative growth and nutrient uptake in several horticultural crops, increasing yield and economic profits to growers [24]. Some authors recommended the use of MLEs as an alternative source for inorganic fertilizers [25]. MLEs were also reported to increase these crucial characteristics under abiotic stress conditions, enabling the plant to maintain growth potential [26,27]. Aqueous MLEs are easy to obtain, inexpensive, and eco-friendly. Leaves are generally abundant in protein, vital amino acids, antioxidants such as ascorbic acid, flavonoids, carotenoids, vitamins A and C, phenolics and macro- and micronutrients [28]. Of course, the composition may vary according to geographic origin and cultivation system. *M. oleifera* is a tree typical of the arid and warm areas of tropical and equatorial regions, but recently started to be cultivated even in Italy in open fields in southern regions with a warm/hot climate or in greenhouses. 

This study aimed to evaluate the effect on lettuce plants grown in a hydroponic cultivation system of aqueous extracts of moringa leaves from plants grown in Apulia as compared to two commercial biostimulants with regard to marketable yield, quality, and storability.

## 2. Results and Discussion

### 2.1. Qualitative and Quantitative Characterization of MLEs

**Figure 1:**
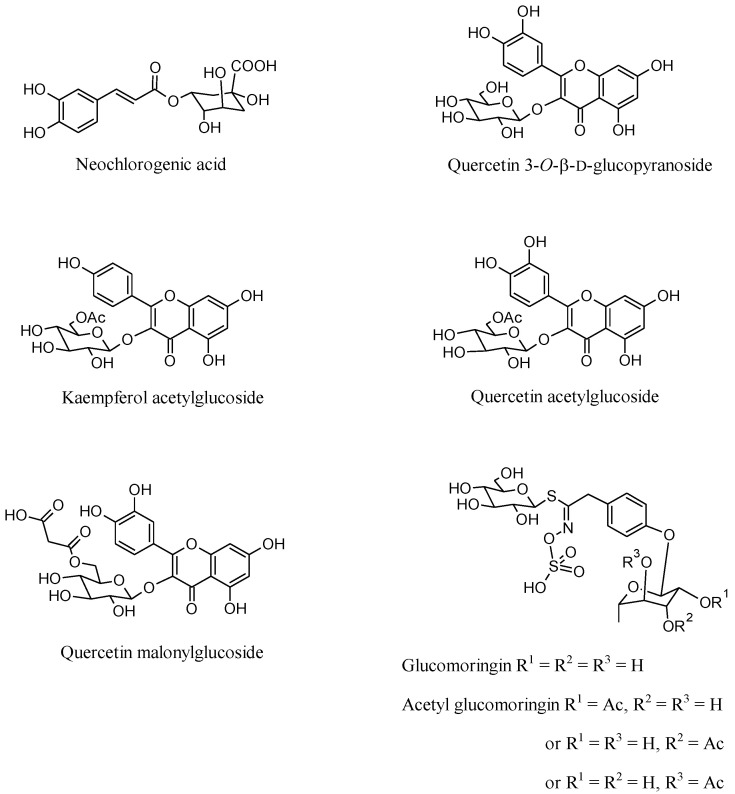
Structures of bioactive compounds tentatively identified in the aqueous extracts of *M. oleifera* leaves (Table 1 and Table 2).

Bioactive compounds from the aqueous extracts of *M. oleifera* leaves obtained from plants cultivated in an open field in the Barletta area (MLEB) and in a greenhouse in Lecce province (MLES), respectively, were tentatively identified through their MS spectra. Only compounds whose deprotonated ion mass differed from their corresponding calculated mass by <5 ppm and displaying fragmentation patterns in agreement with the literature were included in Table 1 and Table 2. Neochlorogenic acid, quercetin 3-*O*-β-D-glucopyranoside, and two typical Moringa secondary metabolites, namely glucomoringin and its acetyl derivative [29], were identified in both tested extracts (Figure 1).

**Figure 2:**
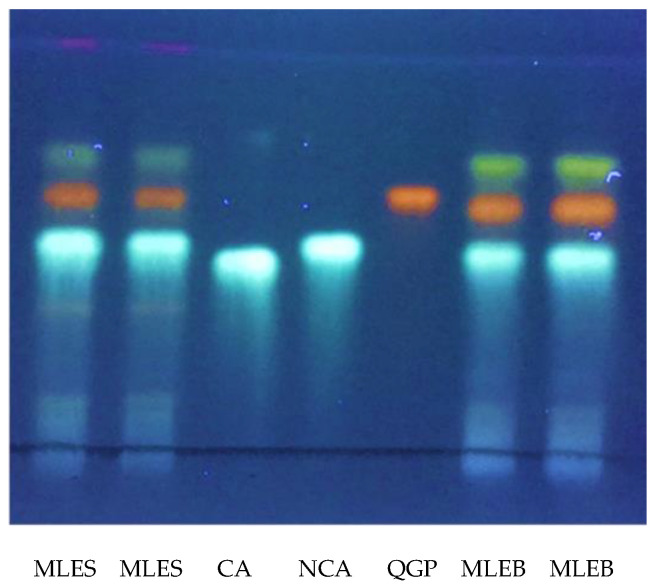
HPTLC profile of both *M. oleifera* leaf water extracts obtained by microwave irradiation (each seeded twice) and three reference standards sprayed with NP/PEG reagent; MLEB: *M. oleifera* Barletta; MLES: *M. oleifera* Salento; CA: chlorogenic acid; NCA: neochlorogenic acid; QGP: quercetin 3-*O*-β-d-glucopyranoside; solvent: ethyl acetate:formic acid:acetic acid:water (100:11:11:26); λ: 366 nm.

The presence of neochlorogenic acid and quercetin 3-*O*-β-d-glucopyranoside were also confirmed by high-performance thin-layer chromatography (HPTLC) analysis using reference standards (Figure 2). HPTLC analysis was also useful to confirm the exact nature of the chlorogenic acid isomer extracted. In fact, chlorogenic and neochlorogenic acids are classical diastereomers that differ in the stereochemistry at positions 1 and 4 of their sugar-like moieties, their corresponding overall stereochemistry notations being (1S,3R,4R,5R) and (1R,3R,4S,5R), respectively. They share the same molecular weight value and, although having quite different mass fragmentation patterns, an accurate and unquestionable identification is advisable. To the contrary, kaempferol acetylglucoside and quercetin acetylglucoside were identified only in MLEB (Table 1). Besides these compounds, MS analysis also revealed the presence in MLEB of the amino alcohol valinol; the organic acids quinic, malic, and gluconic acids; the phenylpropanoids caffeic, coumaroylquinic, and 3-feruloylquinic acids; the flavonoids vitexin, astragalin, quercetin malonylglucoside, kaempferol malonylglucoside, and quercetin 3-[6’’-(3-hydroxy-3-methylglutaryl)glucoside]. Valinol, quinic, caffeic, and coumaroylquinic acids, and astragalin were also detected in MLES, with this latter containing additionally apigenin glucoside and kaempferol diglucoside (Table 2). Flavonol and flavone glycosides occur in common vegetables, mainly as the quercetin or kaempferol glycosides and less frequently as the luteolin or apigenin glycosides [33]. Their formation normally depends on light, so they are mainly concentrated in the outer tissues of the free-standing leaves. Quercetin’s ability to induce resistance phenomena in plant tissues has been reported [34], and the induction of the phenylpropanoid pathway is considered to be involved in resistance mechanisms to stresses [35]. Glucomoringin and its acetyl derivative were reported to play an antioxidant and antimicrobial role [29].

### 2.2. Yield, Morphological Parameter Measurements and Quality Analysis Samplings

**Figure 3:**
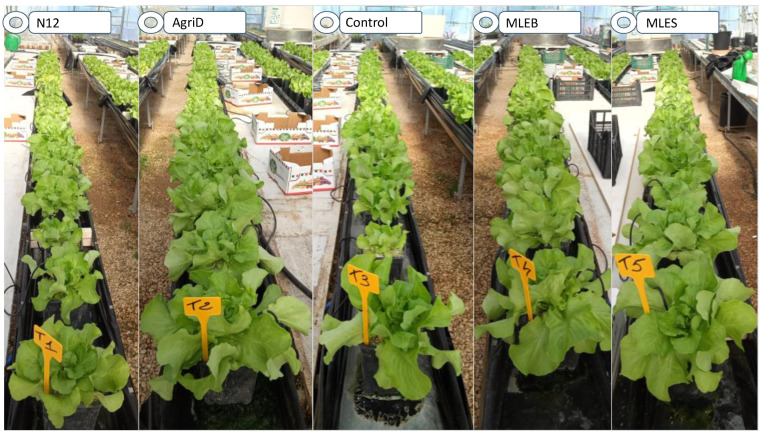
Overview of lettuce plants at harvest time treated with different biostimulants.

Results showed that the application of the commercial formulate based on Fabaceae enzymatic hydrolysate AgricostanD (AgriD) increased the marketable yield, head weight, and leaf area of lettuce plants by 38, 37 and 22% (Table 3, Figure 3), respectively, as compared to the untreated control. 

Similar results were obtained using PHs on spinach [36], tomatoes [37], and wine grapes [38]. In addition, the tested MLEs even improved some agronomic parameters as compared to the control; in particular, the marketable lettuce yield and leaf area were increased by 30 and 14%, respectively, in MLEB-treated samples and the leaf number by 16% in the presence of MLES (Table 3). Other studies also reported that lettuce treated by MLEs showed increased plant growth parameters such as the number of leaves per plant, leaf area and yield [21,39]. Finally, Organor N12 (N12) increased leaf area by 13% as compared to the control, with an effect comparable to MLEB. Whereas, concerning the evaluation of the leaf water content (WC) and the dry matter (DM), no significant differences were observed in treated plants as compared to the control (Table 3). The presence of bioactive peptides/compounds triggering signaling pathways involving phytohormone biosynthesis may explain the positive effects of the tested biostimulants on plant growth and yield [18]. Enhanced growth might also be ascribed to the presence of sugars in the biostimulants, which might represent a source of energy and stimulate nitrogen assimilation [21]. Furthermore, the addition of exogenous amino acids present in PHs might have an energy-saving role, so that the plant can better diverge this energy towards other biological functions [40]. In addition, flavonoids, among the main MLE constituents, have long been known to be responsible for many biological functions, including seedling growth and development [41]. As such, applications of MLEs could contribute to increased vegetative growth as well as nutrient uptake in different horticultural crops, ultimately increasing crop productivity and nutritional values and consequently attracting consumers’ attention [25].

### 2.3. Leaf Color, SPAD Index, and Pigment Contents

Consumer preference is mainly determined by product appearance, which is linked particularly to color [42]. In the current study, all biostimulant treatments, except for MLEB, influenced the leaf hue angle (*h°*), which corresponded to the common distinction of green among the colors (Table 4). The highest *h°* value, as compared to the control, was obtained from samples treated with N12, followed by MLES and AgriD. The increase in hue angle gave lettuce leaves the appearance of a warm green color (Figure 2) that might be attractive to consumers. Concerning the chroma value (C*), indicating the saturation of the color, only MLEB caused a 3% increase (Table 4). In contrast, none of the treatments increased the leaf lightness (L*). 

Finally, the Soil Plant Analysis Development (SPAD) index, a non-destructive indicator of N leaf content, showed a 10% increase in samples treated with N12 (Table 4), a finding which was not surprising given its high N content. Additionally, biostimulant application induced a significant increase in lettuce leaf pigments (Table 4), particularly total chlorophyll and carotenoid contents, reaching the highest increase with AgriD (15 and 16%, respectively), followed by MLES (10 and 11%, respectively). This ability to increase pigment content is in line with other reports on plant-based biostimulants, whose application may lead to the up-regulation of photosynthesis and improved nitrogen and carbon metabolism by enhancing N uptake efficiency and limiting chlorophyll degradation and leaf senescence [43]. Chlorophyll content and photosynthesis rate in plants are known to determine growth and yield. The application of PHs and MLEs are reported to enhance both these vital traits under normal and stress conditions. For instance, the application of MLEs on rocket was reported to increase photosynthetic pigments, photosynthetic rate, and stomatal conductance as compared to untreated plants [21]. In another study, snap bean (*Phaseolus vulgaris*) sprayed with MLE under normal growing conditions showed increased chlorophyll pigments compared to unsprayed plants [26]. The same findings were also reported in the foliar application of PHs in baby rocket leaves and lettuce [43,44], tomato [37], and corn [12].

### 2.4. Total Phenolic Compounds and Antioxidant Activities

**Figure 4:**
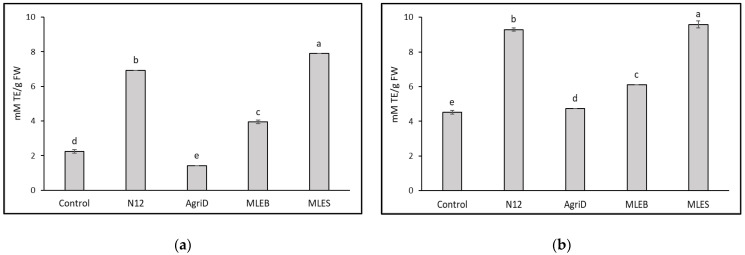
Effect of biostimulant application on lipophilic (DPPH) (**a**) and hydrophilic (ABTS) (**b**) antioxidant activity in lettuce leaves. TE = Trolox Equivalents. Different letters indicate significant differences according to Fisher’s test (*p* ≤ 0.05).

**Figure 5:**
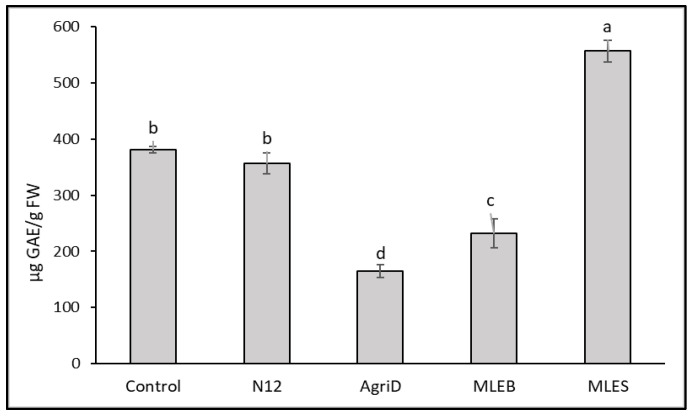
Effect of biostimulant application on total phenolic compounds (TPC) in lettuce leaves. Different letters indicate significant differences according to the Fisher’s test (*p* ≤ 0.05).

The importance of antioxidant activity as a functional quality parameter of food is related to the beneficial effects of antioxidant molecules (hydrophilic and lipophilic) on human health due to their role in delaying or inhibiting oxidative damage, hence evading a broad range of diseases [25]. Leafy vegetables are considered important sources of antioxidant molecules, whose content might be conveniently enriched. In this study, biostimulant application significantly influenced the results from the antioxidant activity evaluation performed by DPPH and ABTS (Figure 4); the highest values were registered in samples treated with MLES, producing a 72 and 53% increase in DPPH and ABTS values, respectively, followed by N12 (68 and 51%), and MLEB (43 and 26%). In contrast, AgriD elicited positive values only for ABTS antioxidant activity. Furthermore, total phenolic compounds (TPC) increased notably, by 34%, in samples treated with MLES as compared to untreated plants (Figure 5). 

In this regard, the application of plant biostimulants, including MLEs, can modify both primary and secondary metabolism, resulting in an increased concentration of antioxidant compounds. For instance, spinach plants supplemented with PHs showed an increase in the concentration of phenolic antioxidants, total soluble proteins, and other bioactive compounds [36]. Selected plant leaf extracts (mulberry, brassica, sorghum and moringa) significantly enhanced the radical scavenging capacity of radish as compared to the control [45]. Finally, MLE alone and in combination with K and Zn improved quality parameters, such as soluble solid contents, vitamin C, sugars, total antioxidant and phenolic contents, and the activities of superoxide dismutase and catalase enzymes in ‘Kinnow’ mandarin fruit [24]. It has been proposed that biostimulants might mimic a stressing event with the consequent induction of the plant responding system, including the antioxidant system, leading to an increase in antioxidant molecule production in plants [38,43].

### 2.5. Resistance to Postharvest Grey Mold

**Figure 6:**
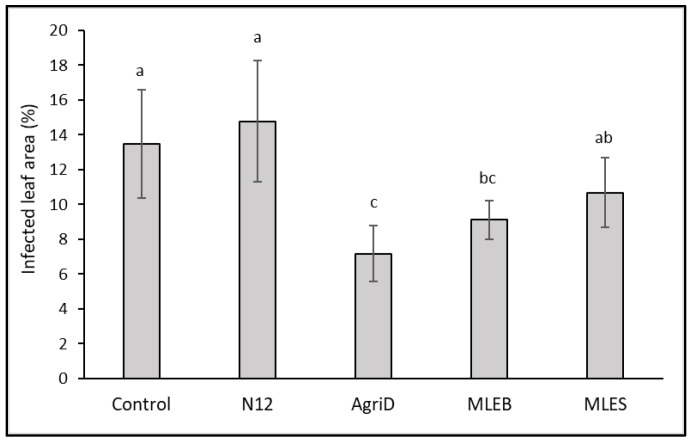
Effect of biostimulant application on infected area (%) of lettuce leaves following *Botrytis cinerea* infection. Different letters indicate significant differences according to Fisher’s test (*p* ≤ 0.05).

In our investigation, we also tested the resistance of treated plants to the economically dangerous fungal pathogen *Botrytis cinerea*, which can compromise yield and quality both during cultivation and after harvest. The lettuce leaves treated with MLEB and AgriD during the growth phase proved to be more resistant to the fungal infection, showing a reduced disease severity by 32 and 46%, respectively, as compared to control leaves (Figure 6). This finding is not surprising as soybean PHs proved to be effective against grey mold and downy mildew [40,46] on grapevines and green mold on citrus fruit [47]. The effect could be related to the induction of defense responses [46], including the closing of stomata [38]. A similar behavior might be supposed for MLEs, and particularly the MLEB, also given the inclusion of molecules such as quercetin and apigenin glucosides that might contribute to resistance to stresses [34,48,49].

## 3. Materials and Methods

### 3.1. Lettuce Growth Conditions, Experimental Design, and Treatments

The study was carried out in a greenhouse at the CIHEAM Bari (Valenzano, Apulia, Italy) in the period March–May 2022. Seeds of lettuce [*Lactuca sativa* L. cv. ABAGO RZ (43-72), Rijk Zwaan, Italy] were sown into seed cells (0.0014 holes/m^2^) filled with a commercial substrate (Brill® 3 Special, Agrochimica, Bolzano, Italy). After 10 days, when lettuce seedlings had fully expanded cotyledons, they were transplanted to individual pots with perlite medium for hydroponic systems. The pots were randomly distributed in 5 rows; each row included 15 pots and was subjected to one treatment. Lettuce seedlings were fertigated using a drip system with a nutrient solution of FloraGrow/Micro and FloraBloom used according to the manufacturer’s instructions (General Hydroponics, Portland, USA). The irrigation frequency was 5 times daily for a total of 1.25 L water/day/plant.

Four treatments were applied: once at the transplanting by root immersion, and then three times at 10-day intervals during the growth period by foliar spray until dripping, using a steel spray bottle. The applied treatments were two commercial plant biostimulants based on blended nitrogen sources (Organor N12, ICAS International, Milan, Italy, 4 mL/L) and a Fabaceae enzymatic hydrolysate (AgricostanD, Costantino Srl, Favria, TO, Italy, 2.5 mL/L) and two aqueous extracts, namely MLES and MLEB, from leaves of *M. oleifera* plants cultivated in a greenhouse in Lecce province (South Apulia, Italy) and open field in Barletta area (North Apulia, Italy), respectively. Plants treated with water were used as a control. 

### 3.2. Moringa Leaves Extract Preparation and Characterization

Based on our previous experience of microwave-assisted extraction of bioactive compounds from different plant matrices [50,51,52,53], the extraction was carried out under microwave irradiation. The fresh moringa leaves were dried before undergoing microwave-assisted extraction. Briefly, in a microwave tube, 200 mg of leaf powder was briefly suspended in 2 mL of bi-distilled water and extracted for 5 min at 80 °C. After filtration, the sample was centrifuged for 10 min at 8000× *g*, and the supernatant was lyophilized and resuspended in 500 mL of bi-distilled water. The extracts were qualitatively characterized using a High-Resolution Mass Spectrometry (HRMS) approach. Analyses were performed using a microTOF QII mass spectrometer (Bruker Daltonics, Bremen, Germany) equipped with ESI operating in both positive and negative ion modes. Bioactive compounds were tentatively identified through their MS spectra in comparison to literature data.

For HPTLC analysis, a mobile phase consisting of ethyl acetate:formic acid:acetic acid:water (100:11:11:26) was used. The application of standards and samples was performed using a semimicro applicator (Cellogel Electrophoresis Company, Milan, Italy). Approximately 1.5 μL of extract sample and 1.5 μL of 1 mg/mL solution of chlorogenic acid (CA), neochlorogenic acid (NCA), and quercetin 3-*O*-β-d-glucopyranoside (QGP) standards were separately applied in the form of bands (1.5 μL × 8 mm) at 1 cm from the bottom using TLC Silica gel 60 F254 pre-coated plate (Merck). The plate was developed up to the distance of 8 cm from the bottom, air dried, heated at 100 °C for 5 min, sprayed with 1% (*w*/*v*) diphenylboryloxyethylamine in methanol (NP), then sprayed with 5% (*w*/*v*) polyethylene glycol 4000 (PEG4000) in ethanol, air dried and visualized by viewing in UV-cabinet under long wavelength (366 nm).

### 3.3. Yield and Agronomic Parameters

The lettuce plants were harvested at commercial maturity. The external leaves were removed, and each head was weighted to determine marketable yield (g) per treatment including the control. Five homogenous plants, located at the center of the row, were selected per treatment for measuring leaf number and area. The leaf area of each plant was estimated using the open access software ImageJ 1.53 version (U.S. National Institutes of Health, Bethesda, MD, USA) and quantified in cm^2^. Samples from the abovementioned plants were put in a forced air-drying oven at 60 °C until a constant weight was reached for the successive determination of WC and DM. The WC was calculated according to the formula (1)

WC = [(FW − DW)/FW] × 100(1)

whereas DM was calculated following Cristofano et al. [54] according to the formula (2)

DM = (leaf dry weight/leaf fresh weight) ×100(2)

For qualitative analysis, per treatment, fresh leaf sub-samples were frozen with liquid N, powdered, and stored at −80 °C.

### 3.4. Leaf Colour, SPAD Index, and Pigment Content

Colour CIELAB parameters (L*, C*, and a*, b*) were measured by a Chromameter (CR-300, Minolta Co. Ltd., Tokyo, Japan) between the midrib and the leaf margin in 10 undamaged lettuce leaves per replicate. The L* coordinate expressed the degree of lightness of the measured color (100 = white; 0 = black). The a* and b* values were used to calculate the hue angle (*h°*) according to the formula (3)

*h°* = tang −1 (b*/a*) (3)

The *h°* expressed the color tones in degrees: 0° (red color), 90°(yellow), 180° (green), and 270° (blue). The C* described saturation of the color: the higher the C* value, the more saturated the color. 

Moreover, the SPAD index, indicating N leaf content, was measured in 10 fully expanded leaves per replicate by SPAD-502Plus (Minolta) following manufacturer specifications. 

Finally, pigment content of the leaves was spectrophotometrically determined [49]. From each treatment, 1 g from a pool of 5 freshly frozen powdered leaves was homogenized with pure acetone (HPLC-UV grade, Pharmco-Aaper, Brookfield, CT, USA), incubated for 15 min on ice, centrifuged for 5 min at 14,000× *g* at 4 °C, filtered with 0.45 µm pore size nylon syringe filters (MilliporeSigma, Bedford, MA, USA), and transferred into spectrophotometric cuvettes. Quantification of chlorophyll and carotenoids was performed using a Cary 60 UV-VIS spectrophotometer (Agilent Technologies, Santa Clara, PA, USA) at three different wavelengths: 662, 645, and 470 nm. The concentrations were calculated according to the following formulas:

Chlorophyll a (Chl_a_) = (11.24 × A_662_) − (2.04 × A_645_) (4)

Chlorophyll b (Chl_b_) = (20.13 × A_645_) − (4.19 × A_662_) (5)

The total chlorophyll was calculated as the sum of Chl_a_ and Chl_b_ (6).

Carotenoids = (1000 × A_470_ − 1.9 Chl_a_ − 63.14 Chl_b_)/214 (6)

### 3.5. Total Phenolic Compounds and Antioxidant Activities

One gram of fresh powdered lettuce leaves was homogenized with 5 mL ethanol 70% and then subjected to ultrasound for 15 min, centrifuged at 9000× *g* and filtered with 0.45 µm pore size nylon syringe filters. The determination of the total phenol content (TPC) was performed using the Folin–Ciocalteu method as described by Tarantino et al. [55] The antioxidant activity was determined using a free DPPH radical (1,1-diphenyl-2-picrylhydrazyl) and ABTS radical (2,20-azino-bis (3-ethylbenzothiazoline-6-sulfonic acid) [56]. To measure the reduction in absorbance of the solutions a spectrophotometric assay was carried out at 517 and 734 nm wavelengths, respectively. The antioxidative activity of lettuce was expressed in mM Trolox Equivalents (TE) per g of fresh lettuce. 

### 3.6. Resistance to Postharvest Grey Mold

A strain (FV52) of *B. cinerea* from the Culture Collection of the University of Bari Aldo Moro (Italy) was grown on PDA at 24 °C with a 14 h/10 h photoperiod for 7 days. Ten leaves per treatment (2 leaves per plant, 5 plants per treatment) were used. They were placed in plastic boxes containing a moistened filter paper with sterile distilled water at the bottom, this latter was topped by a plastic net to separate the leaves from the paper. The leaves were inoculated by depositing a mycelial plug, 5 mm in diameter, from the active growing margins of the *B. cinerea* colony [57]. The boxes were sealed in plastic bags and incubated at 18 °C for 4 days. Finally, leaves were assessed for the infected area as percentage (%) of leaf surface.

### 3.7. Statistical Analysis 

All data were subjected to a one-way analysis of variance (One-way ANOVA) using Minitab 19 (Minitab Inc., State College, PA, USA). Means were separated utilizing Fisher, as a post-hoc test, performed at *p* ≤ 0.05 significance level.

## 4. Conclusions

The present study provides some evidence of the positive effect of biostimulant application on the yield, quality, and storability of greenhouse-grown lettuce. Effective commercial products exist, but a new promising biostimulant could be introduced. *M. oleifera* seems a good candidate as it is already used for human consumption, was recently proposed as a possible candidate for replacing animal proteins and is to be included among basic substances (i.e., active substances, not predominantly used as plant protection products but which may be of value for plant protection and for which the economic interest in applying for approval may be limited, EC Regulation 1107/2009). If included in this list, MLEs could be legally used in the EU. Although further studies on a larger scale and the mode of action are needed, the proposed data seem promising.

## Figures and Tables

**Table 1:** Tentative compounds identified in water extract of *M. oleifera* leaves from Barletta (MLEB) by HRMS under negative ([M − H]^–^) ionization.

Compound	Experimental Mass	Calculated Mass	Key Fragments (*m*/*z*)	Error (ppm) ^a^	Molecular Formula	Reference
Neochlorogenic acid	353.0895	353.0878	135.0449 (100), 179.0346 (27.0), 191.0557 (80.9)	3.4	C_16_H_18_O_9_	[30,31]
Quercetin 3-*O*-β-d-glucopyranoside	463.0886	463.0880	271.0249 (22.3), 300.0300 (100), 301.0354 (52.5)	1.3	C_21_H_20_O_12_	[30,31]
Kaempferol acetylglucoside	489.1046	489.1037	284.0336 (100), 285.0356 (78.6)	1.8	C_23_H_22_O_12_	[30]
Quercetin acetylglucoside	505.100	505.0988	271.0260 (21.9), 300.0294 (100), 301.0358 (55.6)	2.4	C_23_H_22_O_13_	[30,31]
Quercetin malonylglucoside	549.0905	549.0886	300.0271 (100), 301.0335 (56.7)	3.5	C_24_H_22_O_15_	[30]
Glucomoringin	570.0972	570.0957	96.9612 (100), 259.0144 (6.8), 328.0879 (7.1)	2.6	C_20_H_29_NO_14_S_2_	[30,31,32]
Acetyl glucomoringin	612.1075	612.1062	96.9612 (100), 259.0144 (9.59)	2.1	C_22_H_31_NO_15_S_2_	[31]

^a^ calculated at the site https://warwick.ac.uk/fac/sci/chemistry/research/barrow/barrowgroup/calculators/mass_errors/ through the mass, *m*/*z*, and error calculator of the Barrow group, Department of Chemistry, University of Warwick.

**Table 2:** Tentative compounds identified in water extract of *M. oleifera* leaves from Salento (MLES) by HRMS under negative ([M − H]^–^) ionization.

Compound	Experimental mass	Calculated mass	Key fragments (*m*/*z*)	Error (ppm) ^a^	Molecular formula	Reference
Neochlorogenic acid	353.089	353.0878	135.0465 (100), 179.0379 (27.57), 191.0578 (91.93)	3.4	C_16_H_18_O_9_	[30,31]
Quercetin 3-*O*-β-d-glucopyranoside	463.0870	463.0880	271.0244 (18.02), 300.0266 (100), 301.0330 (50.7),	–2.2	C_21_H_20_O_12_	[30,31]
Glucomoringin	570.0955	570.0957	96.9604 (100), 259.0140 (10.33), 328.0866 (8.49	–0.3	C_20_H_29_NO_14_S_2_	[30,31,32]
Acetyl glucomoringin	612.1068	612.1062	96.9606 (100), 259.0118 (7.73)	1.0	C_22_H_31_NO_15_S_2_	[31]

^a^ calculated at the site https://warwick.ac.uk/fac/sci/chemistry/research/barrow/barrowgroup/calculators/mass_errors/ through the mass, m/z, and error calculator of the Barrow group, Department of Chemistry, University of Warwick.

**Table 3:** Effect of biostimulant application on marketable yield and morphological parameters of lettuce.

Treatments	Marketable Yield (g/m)	Head Fresh Weight (g)	Leaf Area (cm^2^)	Leaf Number	Water Content (%)	Dry Matter (%)
Control	799.52	53.30 ± 26.07 b	130.44 ± 40.15 c	21.40 ± 2.19 b	90.63 ± 0.37 ab	9.37 ± 0.37 ab
N12	818.79	54.59 ± 28.73 b	150.73 ± 44.49 ab	20.80 ± 2.05 b	89.35 ± 0.48 b	10.65 ± 0.48 a
AgriD	1280.18	85.30 ± 46.80 a	168.21 ± 32.92 a	23.40 ± 1.14 ab	91.78 ± 1.31 a	8.22 ± 1.31 b
MLEB	1144.91	76.30 ± 43.10 ab	151.60 ± 33.08 ab	22.40 ± 1.82 b	92.20 ± 0.44 a	7.80 ± 0.44 b
MLES	999.90	66.66 ± 24.19 ab	134.59 ± 26.47 bc	25.40 ± 2.70 a	90.78 ± 1.82 ab	9.22 ± 1.82 ab

Data represent means ± standard deviation. Values followed by different letters within each column indicate significant differences according to Fisher’s test (*p* ≤ 0.05).

**Table 4:** Effect of biostimulant application on leaf colour, SPAD index and pigment contents of lettuce.

Treatments	L* (lightness)	C* (Chroma Value)	*h°* (Hue Angle)	SPAD Index	Tot Chlorophyll (a + b) (µg/g FW)	Carotenoid Contents (µg/g FW)
Control	57.91 ± 3.42 ab	35.74 ± 2.64 b	178.77 ± 0.03 b	28.58 ± 0.63 b	19.21 ± 0.18 d	4.29 ± 0.06 c
N12	57.21 ± 2.61 c	31.99 ± 2.69 d	178.79 ± 0.02 a	31.76 ± 1.96 a	20.45 ± 0.06 c	4.87 ± 0.02 b
AgriD	56.34 ± 2.20 b	34.51 ± 1.94 c	178.78 ± 0.01 a	29.84 ± 1.40 ab	22.51 ± 0.04 a	5.13 ± 0.03 a
MLEB	56.09 ± 1.94 b	34.68 ± 2.22 bc	178.78 ± 0.01 a	30.00 ± 1.84 ab	21.40 ± 0.10 b	4.83 ± 0.01 b
MLES	53.30 ± 2.50 a	36.96 ± 2.31 a	178.76 ± 0.02 b	30.32 ± 1.01 ab	12.64 ± 0.14 e	3.24 ± 0.17 d

The presented values are non-transformed means. Data represent means ± standard deviation. Values followed by different letters within each column indicate significant differences according to Fisher’s test (*p* ≤ 0.05).

## Data Availability

The data presented in this study are available within the article.

## Sample Availability

Not available.

## References

[1] Mir S.A., Shah M.A., Mir M.M., Dar B.N., Greiner R., Roohinejad S. (2018). Microbiological contamination of ready-to-eat vegetable salads in developing countries and potential solutions in the supply chain to control microbial pathogens. Food Cont..

[2] FAOSTAT. https://www.fao.org/faostat/en/#data/QCL/.

[3] Kim M.J., Moon Y., Kopsell D.A., Park S., Tou J.C., Waterland N.L. (2016). Nutritional value of crisphead ‘Iceberg’and romaine lettuces (*Lactuca sativa* L.). J. Agric. Sci..

[4] Casati D., Baldi L., Sannino L., Espinosa B. (2016). L’importanza economica del comparto della IV gamma. Le Avversità Degli Ortaggi da Foglia Per la IV Gamma.

[5] Searchinger T.D. (2013). Synergies and Tradeoffs for Small Farmers and Climate Mitigation.

[6] Wheeler T., Von Braun J. (2013). Climate change impacts on global food security. Science.

[7] Al-Chalabi M. (2015). Vertical farming: Skyscraper sustainability?. Sustain. Cities Soc..

[8] Corbo M., Campaniello D., Speranza B., Bevilacqua A., Sinigaglia M. (2015). Non-conventional tools to preserve and prolong the quality of minimally-processed fruits and vegetables. Coatings.

[9] European Biostimulant Industry Council. www.biostimulants.eu.

[10] Mattner S.W., Wite D., Riches D.A., Porter I.J., Arioli T. (2013). The effect of kelp extract on seedling establishment of broccoli on contrasting soil types in southern Victoria, Australia. Biol. Agric. Hort..

[11] Dodd I.C., Ruiz-Lozano J.M. (2012). Microbial enhancement of crop resource use efficiency. Curr. Opin. Biotechnol..

[12] Ertani A., Schiavon M., Muscolo A., Nardi S. (2013). Alfalfa plant-derived biostimulant stimulate short-term growth of salt stressed *Zea mays* L. plants. Plant Soil.

[13] Du Jardin P. (2015). Plant biostimulants: Definition, concept, main categories and regulation. Sci. Hort..

[14] Barbosa G., Gadelha F., Kublik N., Proctor A., Reichelm L., Weissinger E., Wohelleb G.M., Halden R. (2015). Comparison of land, water and energy requirement of lettuce grown using hydroponic vs. conventional agricultural methods. Int. J. Environ. Res. Public Health.

[15] El-Nakhel C., Petropoulos S.A., Pannico A., Kyriacou M.C., Giordano M., Colla G., Troise A.D., Vitaglione P., De Pascale S., Rouphael Y. (2020). The bioactive profile of lettuce produced in a closed soilless system as configured by combinatorial effects of genotype and macrocation supply composition. Food Chem..

[16] Puglisi I., La Bella E., Rovetto E.I., Lo Piero A.R., Baglieri A. (2020). Biostimulant effect and biochemical response in lettuce seedlings treated with a *Scenedesmus quadricauda* extract. Plants.

[17] Lisiecka J., Knaflewski M., Spizewski T., Fraszczak B., Kaluzewicz A., Krzesinski W. (2011). The effect of animal protein hydrolysate on quantity and quality of strawberry daughter plants cv. Elsanta. Acta Sci. Pol. Hortorum Cultus.

[18] Kurepin L.V., Zaman M., Pharis R.P. (2014). Phytohormonal basis for the plant growth promoting action of naturally occurring biostimulators. J. Sci. Food Agric..

[19] Gomiero T. (2016). Soil degradation, land scarcity and food security: Reviewing a complex challenge. Sustainability.

[20] Godfray H.C.J., Beddington J.R., Crute I.R., Haddad L., Lawrence D., Muir J.F., Pretty J., Robinson S., Thomas S.M., Toulmin C. (2010). Food security: The challenge of feeding 9 billion people. Science.

[21] Zulfiqar F., Casadesús A., Brockman H., Munné-Bosch S. (2020). An overview of plant-based natural biostimulants for sustainable horticulture with a particular focus on moringa leaf extracts. Plant Sci..

[22] Younis A., Akhtar M.S., Riaz A., Zulfiqar F., Qasim M., Farooq A., Tariq U., Ahsan M., Bhatti Z.M. (2018). Improved cut flower and corm production by exogenous moringa leaf extract application on gladiolus cultivars. Acta Sci. Pol. Hortorum Cultus.

[23] Merwad A.R.M. (2018). Using *Moringa oleifera* extract as biostimulant enhancing the growth, yield and nutrients accumulation of pea plants. J. Plant Nutr..

[24] Nasir M., Khan A.S., Basra S.A., Malik A.U. (2016). Foliar application of moringa leaf extract, potassium and zinc influence yield and fruit quality of ‘Kinnow’ mandarin. Sci. Hort..

[25] Saini R.K., Sivanesan I., Keum Y.S. (2016). Phytochemicals of *Moringa oleifera*: A review of their nutritional, therapeutic and industrial significance. 3 Biotech.

[26] Howladar S.M. (2014). A novel *Moringa oleifera* leaf extract can mitigate the stress effects of salinity and cadmium in bean (*Phaseolus vulgaris* L.) plants. Ecotox. Environ. Saf..

[27] Latif H.H., Mohamed H.I. (2016). Exogenous applications of moringa leaf extract effect on retrotransposon, ultrastructural and biochemical contents of common bean plants under environmental stresses. S. Afr. J. Bot..

[28] Gopalakrishnan L., Doriya K., Kumar D.S. (2016). Moringa oleifera: A review on nutritive importance and its medicinal application. Food Sci. Hum. Wellness.

[29] Yan G., Liping S., Yongliang Z. (2020). UPLC-Q-Orbitrap-MS2 analysis of *Moringa oleifera* leaf extract and its antioxidant, antibacterial and anti-inflammatory activities. Nat. Prod. Res..

[30] Rodríguez-Pérez C., Quirantes-Piné R., Fernández-Gutiérrez A., Segura-Carretero A. (2015). Optimization of extraction method to obtain a phenolic compounds-rich extract from *Moringa oleifera* Lam leaves. Ind. Crop. Prod..

[31] Xu Y.-B., Chen G.-L., Guo M.-Q. (2019). Antioxidant and Anti-Inflammatory Activities of the Crude Extracts of *Moringa oleifera* from Kenya and Their Correlations with Flavonoids. Antioxidants.

[32] Gao Q., Wei Z., Liu Y., Wang F., Zhang S., Serrano C., Li L., Sun B. (2022). Characterization, Large-Scale HSCCC Separation and Neuroprotective Effects of Polyphenols from *Moringa oleifera* Leaves. Molecules.

[33] Herrmann K. (1988). On the occurrence of flavonol and flavone glycosides in vegetables. Z. Lebens. Unters. Forsch.

[34] Sanzani S.M., Schena L., De Girolamo A., Ippolito A., González-Candelas L. (2010). Characterization of genes associated with induced resistance against Penicillium expansum in apple fruit treated with quercetin. Postharvest Biol. Technol..

[35] Yadav V., Wang Z., Wei C., Amo A., Ahmed B., Yang X., Zhang X. (2020). Phenylpropanoid pathway engineering: An emerging approach towards plant defense. Pathogens.

[36] Dewang S.P. (2021). Influence of Soil-application of Fish-protein Hydrolysate Liquid on Growth and Yield of Spinach (*Spinacia oleracea* L.). Asian J. Dairy Food Res..

[37] Rouphael Y., Colla G., Giordano M., El-Nakhel C., Kyriacou M.C., De Pascale S. (2017). Foliar applications of a legume-derived protein hydrolysate elicit dose-dependent increases of growth, leaf mineral composition, yield and fruit quality in two greenhouse tomato cultivars. Sci. Hort..

[38] Boselli M., Bahouaoui M.A., Lachhab N., Sanzani S.M., Ferrara G., Ippolito A. (2019). Protein hydrolysates effects on grapevine (*Vitis vinifera* L.; cv. Corvina) performance and water stress tolerance. Sci. Hort..

[39] Taha M.A., AM A.E.A., El-Shennawy M.Z. (2020). Effect of some Plant Aqueous Extracts on Lettuce Growth, Chemical Constituents, Yield and Downy Mildew Disease. J. Plant Prod..

[40] Lachhab N., Sanzani S.M., Bahouaoui M.A., Boselli M., Ippolito A. (2016). Effect of some protein hydrolysates against gray mould of table and wine grapes. Eur. J. Plant Pathol..

[41] Samanta A., Das G., Das S.K. (2011). Roles of flavonoids in plants. Carbon.

[42] Kyriacou M.C., Rouphael Y., Colla G., Zrenner R., Schwarz D. (2017). Vegetable grafting: The implications of a growing agronomic imperative for vegetable fruit quality and nutritive value. Front. Plant Sci..

[43] Di Mola I., Ottaiano L., Cozzolino E., Senatore M., Giordano M., El-Nakhel C., Sacco A., Rouphael Y., Colla G., Mori M. (2019). Plant-based biostimulants influence the agronomical, physiological, and qualitative responses of baby rocket leaves under diverse nitrogen conditions. Plants.

[44] Xu C., Mou B. (2017). Drench application of fish-derived protein hydrolysates affects lettuce growth, chlorophyll content, and gas exchange. Horttechnology.

[45] Rizwan A., Bushra S., Munawar I., Muhammad M. (2016). Variation in biochemical and antioxidant attributes of *Raphanus sativus* in response to foliar application of plant leaf extracts as plant growth regulator. J. Genetic Eng. Biotechnol..

[46] Lachhab N., Sanzani S.M., Adrian M., Chiltz A., Balacey S., Boselli M., Ippolito A., Poinssot B. (2014). Soybean and casein hydrolysates induce grapevine immune responses and resistance against *Plasmopara viticola*. Front. Plant Sci..

[47] Lachhab N., Sanzani S.M., Fallanaj F., Youssef K., Nigro F., Boselli M., Ippolito A. (2015). Protein hydrolysates as resistance inducers for controlling green mould of citrus fruit. Acta Hortic..

[48] Hojati M., Modarres-Sanavy S.A.M., Ghanati F., Panahi M. (2011). Hexaconazole induces antioxidant protection and apigenin-7-glucoside accumulation in *Matricaria chamomilla* plants subjected to drought stress. J. Plant Physiol..

[49] Vàsquez H., Ouhibi C., Lizzi Y., Azzouz N., Forges M., Bardin M., Nicot P., Urban L., Aarrouf J. (2017). Pre-harvest hormetic doses of UV-C radiation can decrease susceptibility of lettuce leaves (Lactuca sativa L.) to *Botrytis cinerea* L.. Sci. Hort..

[50] Roselli M., Lovece A., Bruno C., Cavalluzzi M.M., Laghezza A., Mercurio A., Lentini G., Corbo F., la Forgia F., Fontana S. (2015). Antioxidant activity of Uva di Troia Canosina: Comparison of two extraction methods. Clin. Immunol. Endocr. Metab. Drugs.

[51] Caputo L., Quintieri L., Cavalluzzi M.M., Lentini G., Habtemariam S. (2018). Antimicrobial and antibiofilm activities of citrus water-extracts obtained by microwave-assisted and conventional methods. Biomedicines.

[52] Milani G., Curci F., Cavalluzzi M.M., Crupi P., Pisano I., Lentini G., Clodoveo M.L., Franchini C., Corbo F. (2020). Optimization of microwave-assisted extraction of antioxidants from bamboo shoots of *Phyllostachys pubescens*. Molecules.

[53] Cavalluzzi M.M., Lamonaca A., Rotondo N.P., Miniero D.V., Muraglia M., Gabriele P., Corbo F., De Palma A., Budriesi R., De Angelis E. (2022). Microwave-Assisted Extraction of Bioactive Compounds from Lentil Wastes: Antioxidant Activity Evaluation and Metabolomic Characterization. Molecules.

[54] Cristofano F., El-Nakhel C., Pannico A., Giordano M., Colla G., Rouphael Y. (2021). Foliar and root applications of vegetal-derived protein hydrolysates differentially enhance the yield and qualitative attributes of two lettuce cultivars grown in floating system. Agronomy.

[55] Tarantino A., Difonzo G., Lopriore G., Disciglio G., Paradiso V.M., Gambacorta G., Caponio F. (2020). Bioactive compounds and quality evaluation of ‘Wonderful’pomegranate fruit and juice as affected by deficit irrigation. J. Sci. Food Agri..

[56] Difonzo G., Aresta A., Cotugno P., Ragni R., Squeo G., Summo C., Massari F., Pasqualone A., Faccia M., Zambonin C. (2021). Supercritical CO_2_ extraction of phytocompounds from olive pomace subjected to different drying methods. Molecules.

[57] Bardin M., Comby M., Lenaerts R., Nicot P.C. (2013). Diversity in susceptibility of *Botrytis cinerea* to biocontrol products inducing plant defence mechanisms. IOBC-WPRS Bull..

